# Socioeconomic and environmental determinants of asthma prevalence: a cross-sectional study at the U.S. County level using geographically weighted random forests

**DOI:** 10.1186/s12942-023-00343-6

**Published:** 2023-08-10

**Authors:** Aynaz Lotfata, Mohammad Moosazadeh, Marco Helbich, Benyamin Hoseini

**Affiliations:** 1 Department of Pathology, Microbiology, and Immunology, School of Veterinary Medicine, University of California, Davis, CA USA; 2 Integrated Engineering, Department of Environmental Science and Engineering, College of Engineering, KyungHee University, Yongin, 446-701 Republic of Korea; 3 Department of Human Geography and Spatial Planning, Faculty of Geosciences, University Utrecht, Utrecht, The Netherlands; 4 Pharmaceutical Research Center, Pharmaceutical Technology Institute, Mashhad University of Medical Sciences, Mashhad, Iran; 5 Department of Medical Informatics, Faculty of Medicine, Mashhad University of Medical Sciences, Mashhad, Iran

**Keywords:** Asthma prevalence, Risk factors, Geographically weighted modeling, Explainable machine learning, Geospatial artificial intelligence

## Abstract

**Background:**

Some studies have established associations between the prevalence of new-onset asthma and asthma exacerbation and socioeconomic and environmental determinants. However, research remains limited concerning the shape of these associations, the importance of the risk factors, and how these factors vary geographically.

**Objective:**

We aimed (1) to examine ecological associations between asthma prevalence and multiple socio-physical determinants in the United States; and (2) to assess geographic variations in their relative importance.

**Methods:**

Our study design is cross sectional based on county-level data for 2020 across the United States. We obtained self-reported asthma prevalence data of adults aged 18 years or older for each county. We applied conventional and geographically weighted random forest (GWRF) to investigate the associations between asthma prevalence and socioeconomic (e.g., poverty) and environmental determinants (e.g., air pollution and green space). To enhance the interpretability of the GWRF, we (1) assessed the shape of the associations through partial dependence plots, (2) ranked the determinants according to their global importance scores, and (3) mapped the local variable importance spatially.

**Results:**

Of the 3059 counties, the average asthma prevalence was 9.9 (standard deviation ± 0.99). The GWRF outperformed the conventional random forest. We found an indication, for example, that temperature was inversely associated with asthma prevalence, while poverty showed positive associations. The partial dependence plots showed that these associations had a non-linear shape. Ranking the socio-physical environmental factors concerning their global importance showed that smoking prevalence and depression prevalence were most relevant, while green space and limited language were of minor relevance. The local variable importance measures showed striking geographical differences.

**Conclusion:**

Our findings strengthen the evidence that socio-physical environments play a role in explaining asthma prevalence, but their relevance seems to vary geographically. The results are vital for implementing future asthma prevention programs that should be tailor-made for specific areas.

**Supplementary Information:**

The online version contains supplementary material available at 10.1186/s12942-023-00343-6.

## Highlights

- Asthma risk and protective factors were assessed with explainable geospatial machine learning.
- The geographically weighted random forest outperformed its conventional aspatial counterpart.
- We found striking non-linear environment-asthma associations.
- Smoking and depression were the most influential risk factors; green space was among the least.
- The relevance of socioeconomic and environmental factors varied geographically.

**Supplementary Information:**

The online version contains supplementary material available at 10.1186/s12942-023-00343-6.

## Introduction

Asthma, a chronic inflammatory airway disease, is among the highest disease burdens globally, with an estimated 262 million people worldwide diagnosed in 2019 [1]. In the United States, approximately 25 million adults have asthma. This equals about 1 in 13 people [2]. Notably, the number of asthmatics is expected to rise further [2], calling for a better understanding of the risk and protective factors and the geographic variation in asthma risk.

“Besides aggregated area-level characteristics (e.g., ethnicity, age, and smoking) associated with asthma prevalence and asthma-related health outcomes [3–6], there is tentative evidence that also socioeconomic and environmental determinants are at play [7, 8]. In the United States, for example, a yearly family income of less than $50,000, a lack of a high school education, and living in high-poverty areas were all connected to an increased risk of asthma [9]. Asthma and allergy disorders are disproportionately more common in minority racial/ethnic groups and those with low socioeconomic levels. Asthma frequency and severity are highest among Puerto Ricans (19.2%), American Indians/Alaska Natives (13%), and Black Americans (12.7%) in the United States, and greater in families living below the poverty line than those living above it (11% versus 8%-9%). Besides, asthma risk was associated with air pollutants (ozone [O_3_], Carbon monoxide [CO], Nitrogen Dioxide[NO_2_], Sulfur dioxide[SO_2_], particulate matter [PM_10_], and particulate matter [ PM_2.5_]) ([10–12]), intense vegetation [13, 14], climatic factors (e.g., rainfall, temperature, humidity, pressure, and wind speed) [15, 16], and distance to industrial corridors and streets (e.g., [17]). Further, it is debated among health professionals whether people’s underlying health conditions (e.g., obesity and mental illness) also relate to asthma [18–22]. However, the empirical evidence concerning asthma’s socioeconomic and environmental determinants remains inconclusive, and the results are partly contradictory.

Previous studies on asthma-environment associations were methodologically limited in two ways. First, we argue that the mixed results originate partly due to the application of conventional linear regression models [23–25]. Lacking theoretical support [26, 27], such linear models do not account for variable interactions, non-linearities, etc. To overcome these deficits, data-driven machine learning models hold promise for environmental health research and have recently emerged as alternatives [28]. While the repertoire of machine learning algorithms is extensive [29, 30], tree-based approaches (e.g., random forest [RF]) can deal with numerous (possibly interacting) covariates, can incorporate non-linear associations, and do not rely on restrictive distributional assumptions of the input data [31]. The random forest algorithm is a powerful ensemble learning method to address both classification and regression problems [32]. In our study we used it for the latter. During the learning process, the algorithm minimizes residual sum of squares. Hastie et al. [33] provide an in-depth discussion and a software implementation is provided by Wright et al. [34]. That said, there is no need to stratify the outcome variable into classes. The random forest algorithm also does not rely on restrictive model assumptions compared to ordinary least squares (OLS). Regression models fitted through OLS assume spatially uncorrelated residuals, homoscedasticity, and normally distributed residuals to be the best linear unbiased estimator.

Second, previous studies applied global regressions to model asthma-environment associations [35]. This practice is problematic, especially when the study area is large, because global models assume that the estimated coefficients are spatially stationary (i.e., they do not change across space regardless of the location) [36]. While there is no plausible reason for such a simplification, the novel geographically weighted random forest (GWRF) model [37] relaxes this constrain, as demonstrated in a few studies [37, 38]. Razavi-Termeh et al. [17] used GWRF to predict asthma associated with a wide variety of environmental data, such as PM_2.5_, ozone (O_3_), and humidity in Tehran, Iran. Grekousis et al. [37] applied GWRF to predicting COVID-19 death rates using socioeconomic and underlying health factors in US counties. Similarly, Quiñones et al. [39] predicted spatial heterogeneity of type 2 diabetes mellitus (T2D) prevalence in the USA using socioeconomic US census data.

This flexible machine learning-based algorithm models spatial heterogeneity in asthma prevalence while accounting for the non-linear relationships and captures location-specific variable importance. Additionally, area-level asthma data are likely spatially patterned [40]. Such spatial correlations are explicitly integrated into the GWRF. Model comparisons between the GWRF and conventional (local) regressions favor the former [37], but we are unaware of a study applying this approach to assess asthma-environment associations.

To respond to both research gaps, the overall aim of our study was to evaluate the associations between asthma prevalence and numerous socioeconomic and environmental risk and protective factors in an ecological study at the county level in the United States. Additionally, we assessed the overall importance of these socioeconomic and environmental factors and how the relative importance varies geographically. Our place-based insights are valuable for planning and sustaining healthcare strategies for vulnerable populations.

## Materials and method

### Study design and population

We obtained cross-sectional data on asthma rate per 100,000 population for all 3059 census counties in the United States. Data were acquired through the Behavioral Risk Factor Surveillance System (BRFSS), an annual statewide sampling telephone inquiry. Eligible respondents were (a) aged at least 18 years and (b) living in a noninstitutionalized household. Respondents were randomly sampled from the target population and interviewed via their phones. On average, 400,000 adults were interviewed each year between 347,000 and 506,000 to measure prevalence [41]. The average size of a county is 967 square miles (standard deviation [SD] ± 1247), with an average population of 103,772. While small in size, we deemed counties a suitable analytical scale while facilitating nationwide analyses.

### Asthma prevalence as the outcome variable

We used new-onset and exacerbation asthma prevalence reported as a percentage of cases per 100,000 people for each county as our outcome variable. Asthma-related information was self-reported using telephone surveys. Adults were asked whether they were ever diagnosed with asthma by a health professional and still have asthma. Responses who answered with “don’t know” or “refused” to answer were excluded and not considered in the national estimates [41]. BRFSS uses person-level survey weights to estimate asthma prevalence, as the Additional file 1: Text indicates. We used the aggregated person-level survey weights per county by BRFSS when we fitted the RF model.

### Covariates

We assessed eleven environmental and 5-year decennial social factors that have been shown to be associated to asthma prevalence. The social covariates focus on adults [12, 42–46]. First, we obtained data on area-level poverty from the American Community Survey [47]. The poverty rate is based on the income-to-poverty ratio, a measure of the annual total family income (adjusted for family size) divided by the poverty guidelines varying by state. The impoverished may face financial barriers that prohibit them from accessing basic healthcare and purchasing medication [48]. Second, proficiency in the English language was also obtained through the American Community Survey [47]. To measure people’s ability to speak English, they ask questions about whether a person speaks a language other than English at home, what language he/she speaks, and how well he/she speaks English. The total number of people with limited English proficiency is divided by the total population. Third, we obtained data from the American Community Survey on minorities capturing the proportions of all populations except white and non-Hispanic to the corresponding population of adults 18 years and older. Fourth, the proportion of total uninsured people (i.e., people with no health insurance or health coverage plan) to the population in each census county was collected [47]. Asthma is widespread among minorities and persons who lack the linguistic skills to explain their symptoms [49], and various research (e.g., [50]) confirm the importance of insurance in asthma control.

Fifth, we included smoking prevalence, defined as people who reported smoking at least 100 cigarettes during their lifetime and who, at the time they participated in a survey, reported smoking every day or some days [51]. Sixth, depression prevalence was measured through the Patient Health Questionnaire, a nine-item depression-screening instrument that asks about the frequency of symptoms of depression in the past two weeks. The four response categories ranged from “not at all” to “nearly every day”. Summary scores ranged from 0 to 27. Depression was defined using a score of ≥ 10 [52]. Seventh, data on obesity prevalence (i.e., the percentage of cases per 100,000 people) was provided by the BRFSS [53]. Self-reported height and weight data were used for the body mass index calculations [53]. Unhealthy behavior such as smoking is linked to an increase in asthma rates [54], as are underlying health conditions such as obesity prevalence ([22]) and depression [55]. Eight, green space was captured through the Normalized Difference Vegetation Index (NDVI) obtained from Google Earth Engine based on Landsat 8 imagery. The NDVI ranges from − 1 to + 1 where positive values refer to more vegetation. Ninth, we used secondary data to capture air pollution estimates. Air pollution data were obtained from U.S. EPA regulatory air monitors. At locations without PM_2.5_ measurements, PM_2.5_ concentrations were estimated using land use regression model and data (e.g., roads, elevation, urbanicity) complemented with satellite-derived air pollution estimates [56]. Tenth, we used annual ozone (O_3_) concentration (ppb) estimates from the v1 empirical models [56, 57]. Finally, we included annual mean air mean temperature (℃) from Oregon State University’s Parameter-elevation Regressions on Independent Slopes Model [58]. Environmental factors such as PM_2.5_ and O_3_ concentrations, as well as green space, have been associated to an increase in asthma rates in various studies (e.g., [17, 59]). In addition, the relationship between temperature and its impacts on asthma rates has received public attention in recent years (e.g., [60]).” The area-level data were linked through a unique identifier. To process the raster layers, we computed the mean values of the pixels within an area.

### Methods

#### Descriptive and exploratory analysis

We used summary statistics to describe the data. We also used Pearson correlations to assess covariate multicollinearity. Correlations above |0.8| were deemed critical [61]. However, none of the bivariate correlations has reached this threshold value. We applied the Moran’s *I* statistic for exploratory spatial analysis of our response variable. A positive Moran’s *I* value refers to positive spatial autocorrelation, a negative one to negative spatial autocorrelation, while values around zero indicate a spatially random pattern. Statistical significance was tested through 999 Monte Carlo simulations [62]. For our analytical assessment we used a row standardized queen’s contiguity. This definition of the weight matrix is predominantly used in several area-level studies [63, 64]. As a sensitivity test, we refitted our models with other weight specifications (i.e. rook’s case [65]) and the results were robust.

A random forest (RF) is a regression-based approach based on ensemble learning [32]. The algorithm comprises many regression trees grown to maximum size without pruning. Each tree is based on a bootstrap sample of the input data; at each node, only a subset of the covariates is selected randomly. The final predictions are obtained by averaging the predictions from the individual trees. Unlike traditional regression, the RF models complex associations, incorporates variable interaction, and does not rely on strict statistical assumptions. We used power transformations to achieve more Gaussian-like distributions of the covariates [66, 67].

Despite model comparisons have revealed that the RF model performs well, particularly on a moderately sized dataset compared to alternative algorithms [32], the algorithm by design does not explicitly account for spatial variation in the regression function. To relax RF’s stationarity assumption, we also fitted a geographical weighted random forest (GWRF) to assess spatial non-stationarity between asthma prevalence and the covariates [68].

Technically, GWRF is a locally calibrated RF based on a moving window approach. It includes only nearby observations using a spatial kernel and a spatial weights matrix [69]. Because our input data (i.e., the centroid of each county) were unevenly distributed across space, our GWRF was set up using an adaptive spatial kernel [68]. Thus, if the observations are more spatially dispersed, the bandwidth will be larger and vice versa. We minimized the out-of-bag (OOB) error to determine an optimal bandwidth. A GWRF has a set of hyperparameters that need to be tuned. Following in the footsteps of others [37, 38], we used Random Grid Search (RGS) on the RF model to optimize the hyperparameters of GWRF ('number of variables randomly sampled' and 'the number of trees') (using CARET library in R). The proportion of randomly sampled features at each node ranged from 1 to 7, and the number of trees ranged from 200 to 1000. We then kept these hyperparameters fixed on GWRF and used the tenfold cross-validation method to select the best bandwidth values (from a set of possible bandwidth values) and chose the one with the highest OOB R^2^. In addition, we set the weight (Weighted = True of the ranger R package) to weight each observation in the local data set. As performance metrics, we used the mean square error (MSE), mean absolute error (MAE), root-mean-square error (RMSE), and coefficient of determination (*R*^*2*^). We then used the Moran’s *I* statistic to assess residual spatial autocorrelation. An in-depth description of the GWRF is provided elsewhere [68, 69].

#### Explainable machine learning

Machine learning algorithms are typically a black-box with no straightforward model interpretation. To enhance the interpretability of the GWRF, we implemented numerous strategies from explainable machine learning [70]. First, we used partial dependence plots to characterize the directions and shapes of each association while accounting for the average effects of the other covariates [71, 72]. Second, we used the global permutation feature importance to evaluate each covariate’s role. The measure ranks the covariates by randomly permuting the covariate values. The larger the loss in model performance when using the permuted covariate, the more important the covariate is deemed to be [32]. Third, GWRF also provides a local feature importance measure. Similar to the permutation-based feature importance of a conventional RF, local feature importances are available in GWRF. In both GWRF and RF, the increase in mean square error (IncMSE) is determined to rank the variables [68]. Mapping the local variable importance allows us to examine how, where, and to what extent each variable affects the outcome geographically [37]. The analyses were conducted in the R Statistical Computing Environment (R Core Team [73]) using the “randomForest” and “SpatialML” packages [68]. For cartography purposes, we used ArcGIS 10.8.1.

## Results

### Descriptive and exploratory assessment

**Fig. 1:**
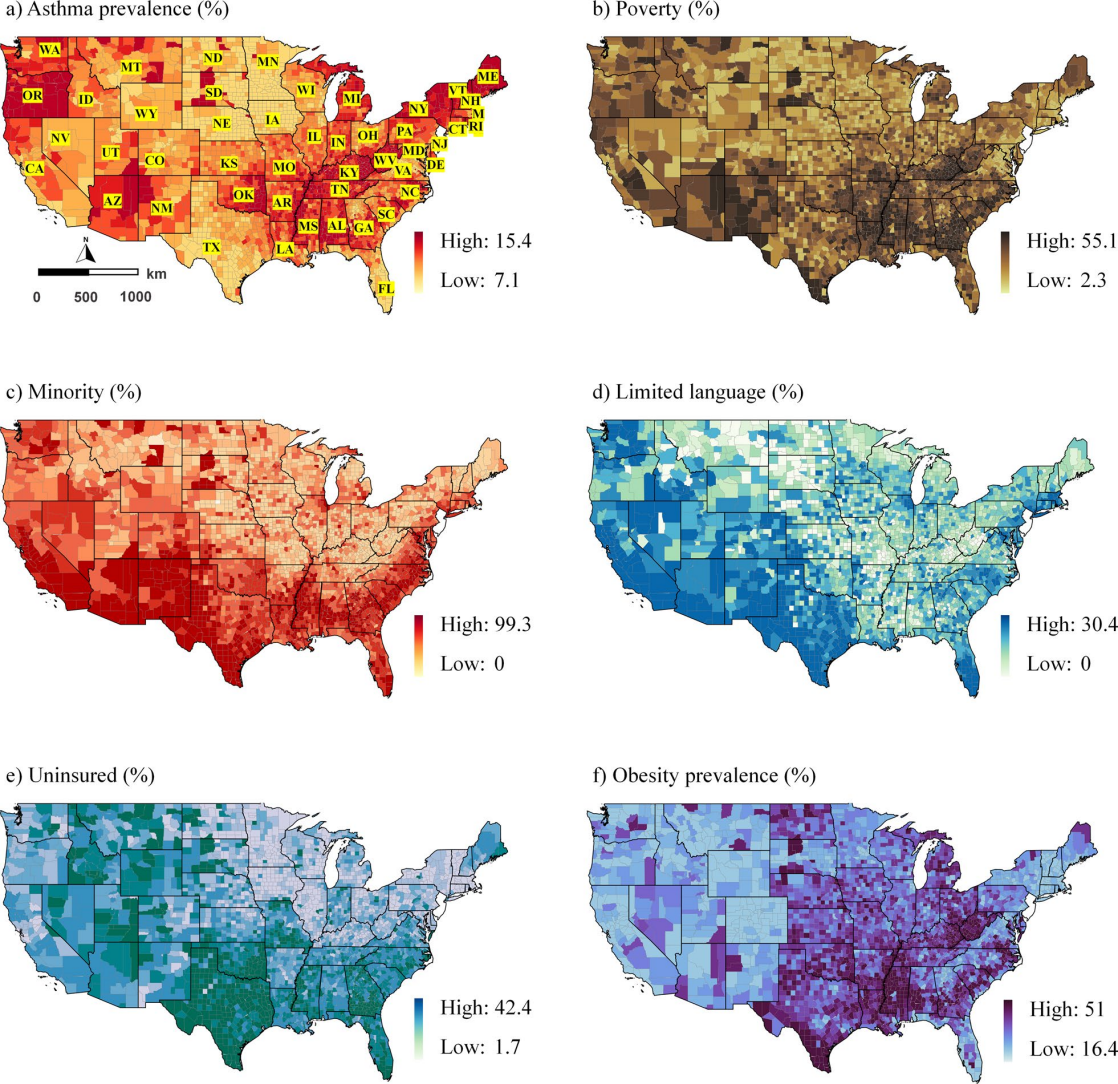

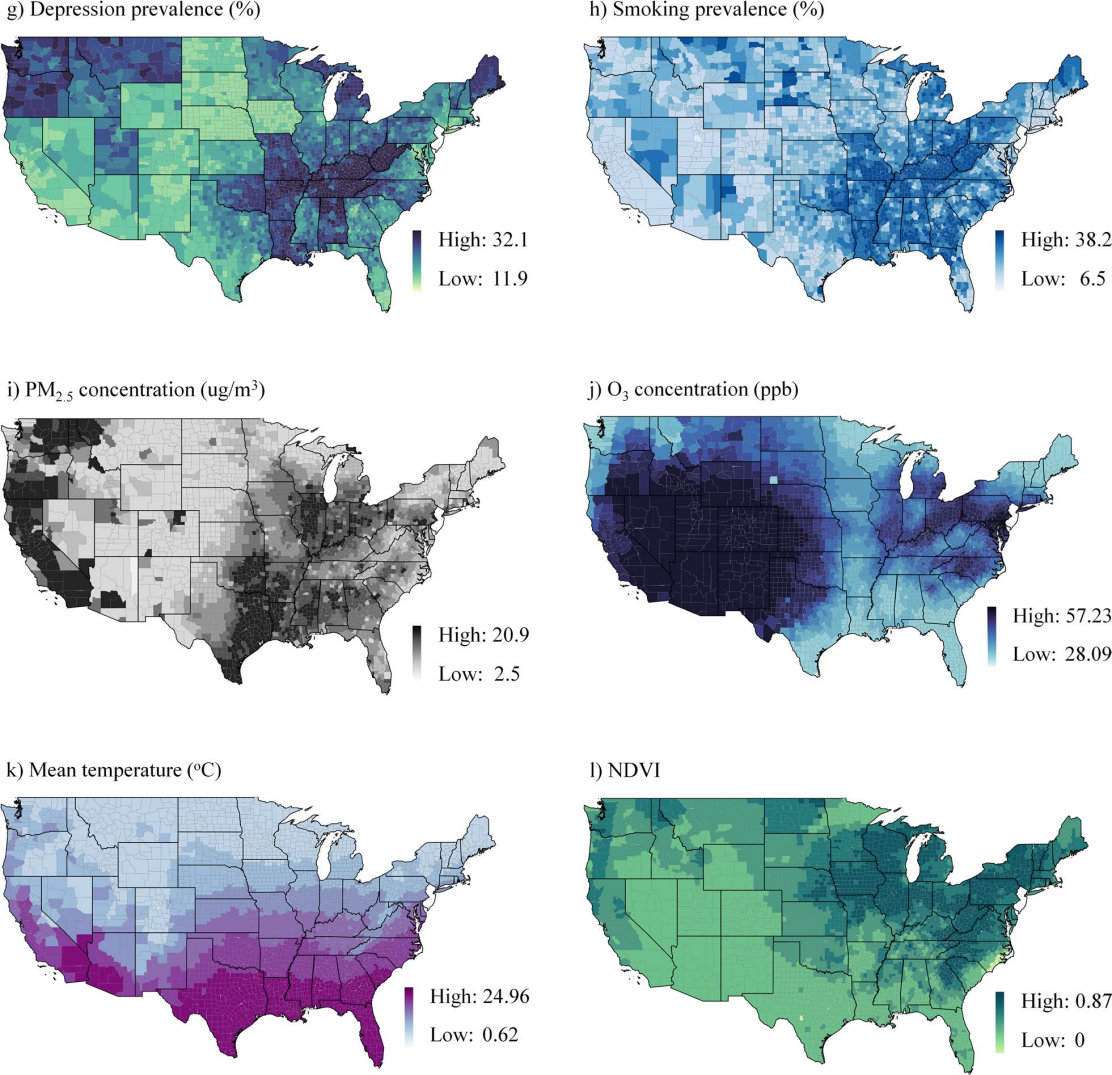
Spatial distribution of the data at the county level; **a**) Asthma prevalence (%) **b**) Poverty (%) **c**) Minority (%) **d**) Limited language (%) **e**) Uninsured (%) **f**) Obesity prevalence (%) **g**) Depression prevalence (%) **h**) Smoking prevalence (%) **i**) PM_2.5_ concentration (ug/m^3^) **j**) O3 concentration (ppb) **k**) Mean temperature (°C) **l**) NDVI (Normalized difference vegetation index).

The untransformed median asthma rate per 1000 persons per area was 9.9, with a standard deviation (SD) of ± 0.99 and an interquartile range of 9.2 and 10.6. Figure 1 illustrates the spatial distribution of the data. Geographically, the asthma prevalence was highest in the northwestern, northwest, a few southwest and northeast counties (Fig. 1a). The impression of spatially autocorrelated asthma prevalence values was supported by a significant Moran’s *I* statistic (*I* = 0.5, *p* < 0.001).

While poverty is distributed unevenly across counties in the United States, minority rates were highest in southern counties (Fig. 1). People with limited English proficiency were concentrated in counties in the west and southwest. The uninsured were prevalent in the southern counties. While the Midwest had the highest prevalence of obesity, the northwestern and midwestern counties had the highest prevalence of depression. Smoking was, however, prevalent in midwestern counties. PM_2.5_ concentrations were most substantial in western counties, whereas O_3_ concentrations were predominantly high across counties, except for a few places in the Midwest. Temperatures in the southern counties were relatively high than in the northern counties. The greenest counties were in the Midwest, North Midwest, and Northeast (Fig. 1). Additional file 1: Table S1 contains additional descriptive information.

### Geographically weighted random forest

#### Model fits

There was no indication of pronounced covariate multicollinearity. As shown in Additional file 1: Table S1, all correlation coefficients were below our a priori-defined threshold value of |0.8|. The tenfold cross-validation suggested that a bandwidth of 108 observations, 1000 trees, and five randomly sampled variables at each split had the highest prediction accuracy. An initial comparison with a traditional RF indicated that our GWRF resulted in lower cross-validated prediction errors (Table 1). In contrast to the non-spatial RF, residuals were spatial uncorrelated in the GWRF. The local *R*^*2*^ of the GWRF varied between 0.22 and 0.95, with an average of 0.31. The model tended to fit better in the north Midwest counties and some places in the eastern counties. Additional file 1: Fig. S1 shows the mapped local *R*^*2*^s.

**Table 1:** Cross-validated prediction accuracy

	RF	GWRF
MSE	17.62	10.61
MAE	3.02	2.80
RMSE	4.20	3.20
*R*^*2*^	0.82	0.89
Moran’s *I* (residuals)	0.28 (*p* < 0.05)	0.0008 (*p* > 0.05)

#### Non-linear associations

**Fig. 2:**
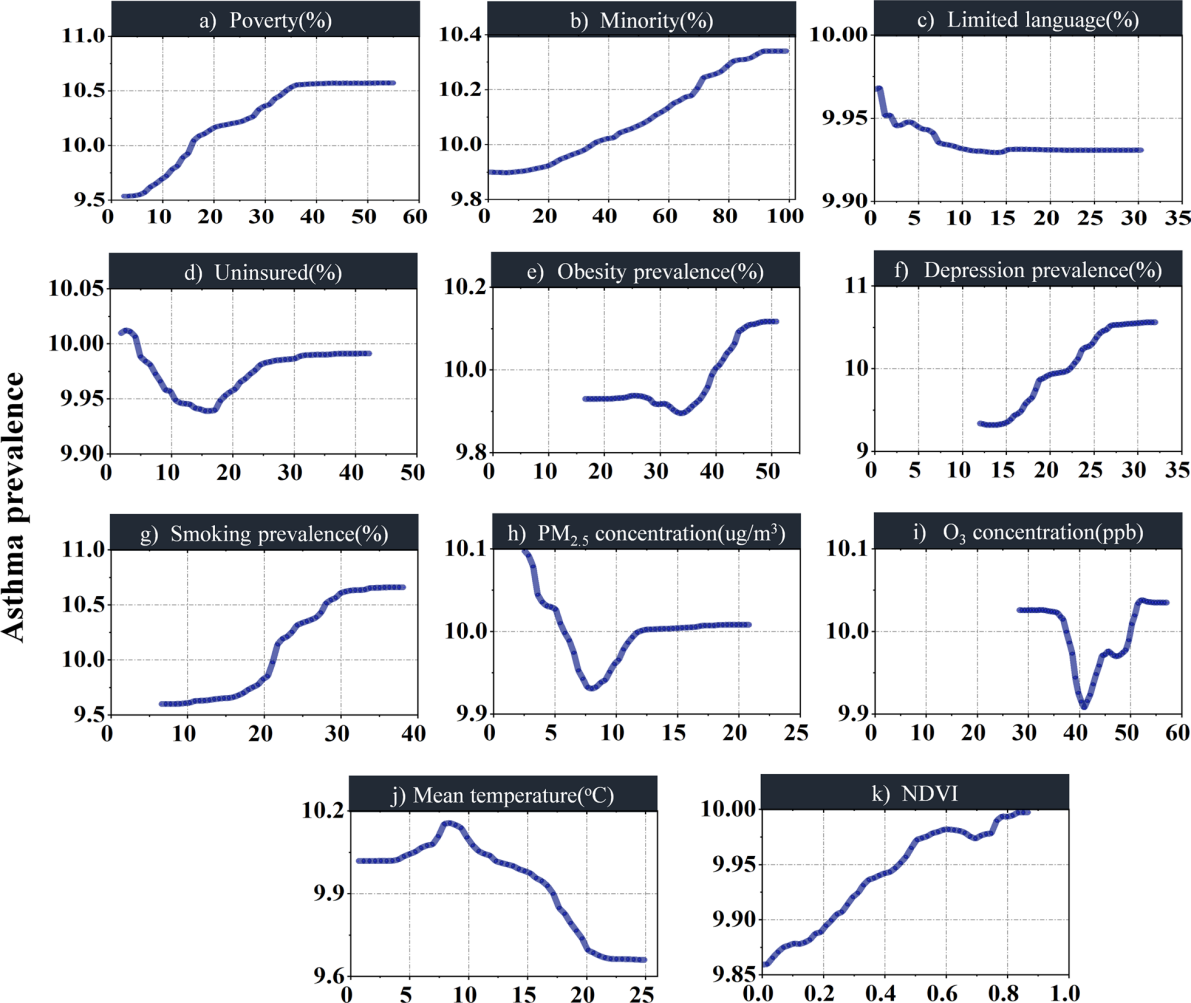
Partial dependence plots based on the RF (The y axis represents asthma prevalence, while the x axis represents asthma determinants); **a**) Poverty (%) **b**) Minority (%) **c**) Limited language (%) **d**) Uninsured (%) **e**) Obesity prevalence (%) **f**) Depression prevalence (%) **g**) Smoking prevalence (%) **h**) PM2.5 concentration (ug/m3) **i**) O3 concentration (ppb) **j**) Mean temperature (°C) **k**) NDVI (Normalized difference vegetation index).

Figure 2 depicts the relationships between asthma prevalence and the covariates as partial dependence plots. We observed that most associations were non-linear and had complex shapes. Some linear correlations only existed within specific variable ranges. Asthma prevalence was positively associated with poverty and minority status but not with limited language skills or being uninsured. Meanwhile, obesity, depression, and smoking were all associated with an increased risk of asthma. There were, however, inverse relationships between asthma prevalence and PM_2.5_, O_3_, and mean temperature. Furthermore, asthma prevalence was positively associated with NDVI.

#### Variable importance

**Fig. 3:**
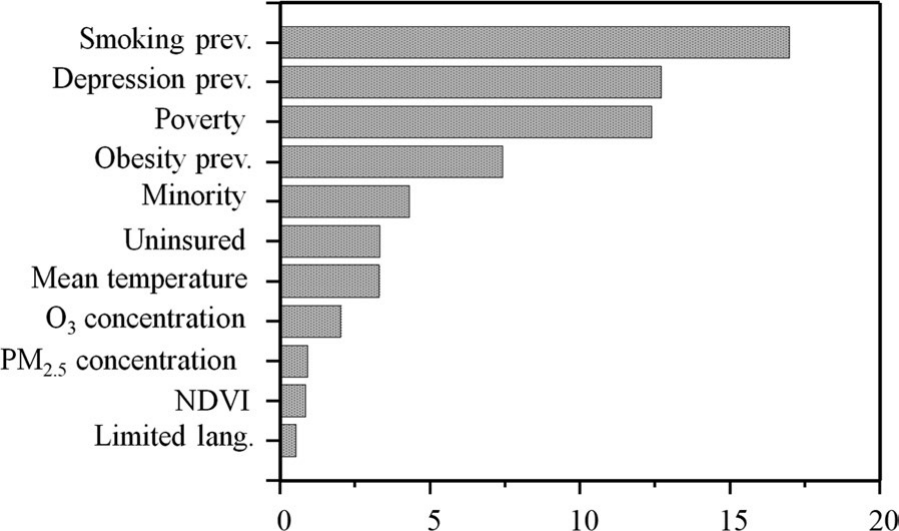
Mean Variable importance based on the GWRF

Figure 3 ranks the importance of covariates in the GWRF model according to the global permutation-based feature importance. The results indicate that smoking is most critical to explain asthma prevalence, followed by depression, poverty, obesity, and minority. Others (e.g., NDVI) play a minor role.

**Fig. 4:**
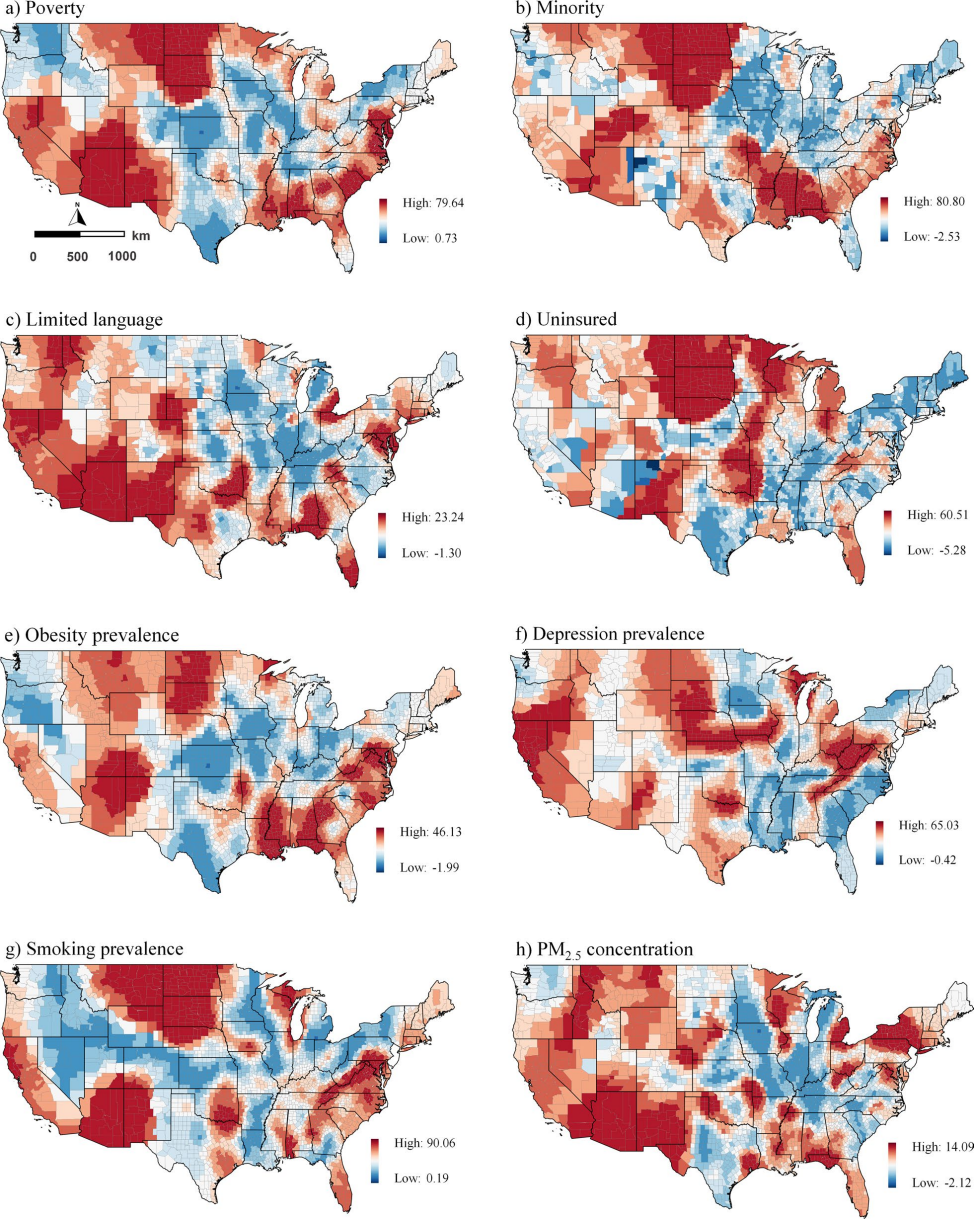

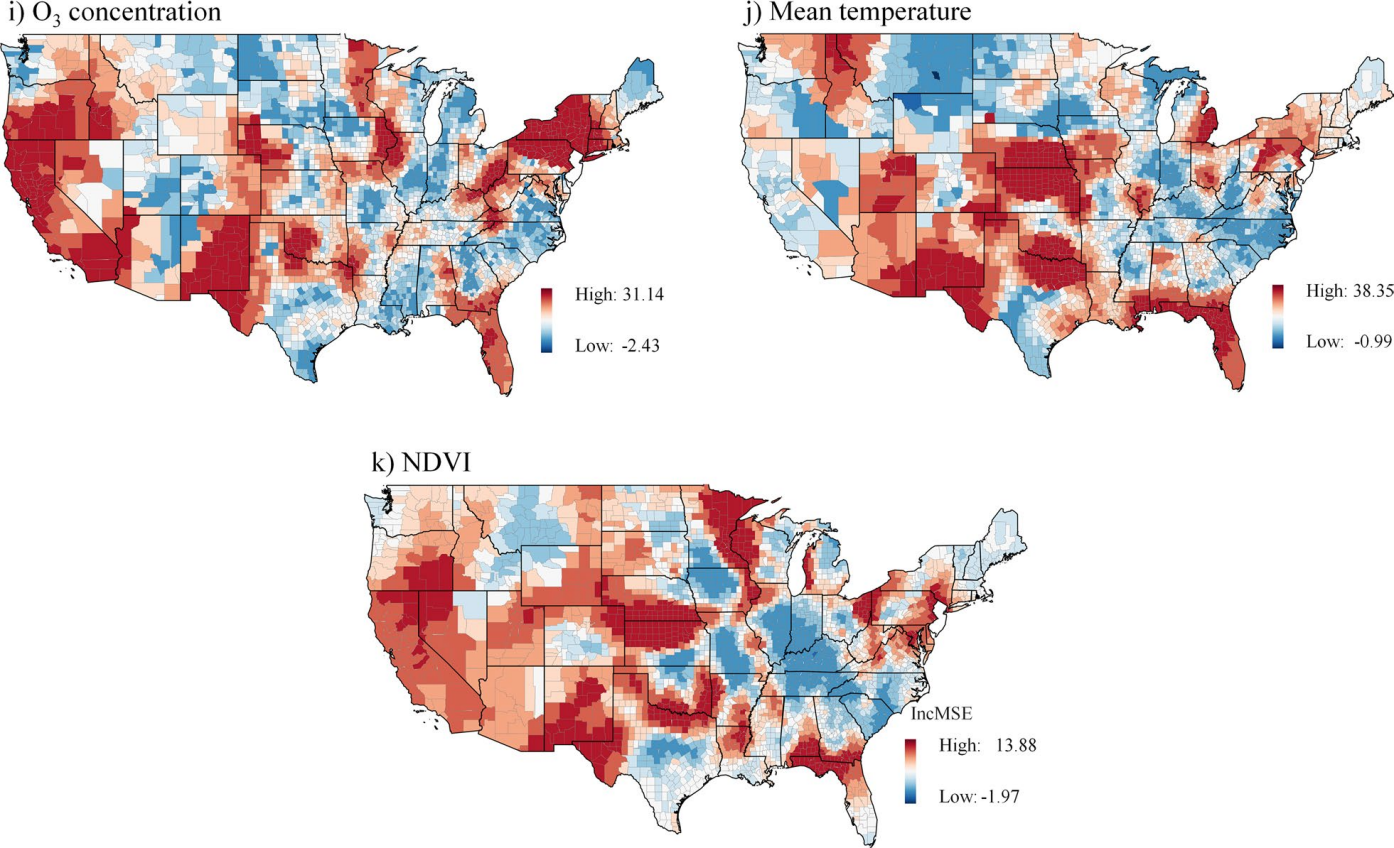
Spatial variation of the local feature importance. Higher values indicate increased importance; **a**) Poverty (%) **b**) Minority (%) **c**) Limited language (%) **d**) Uninsured (%) **e**) Obesity prevalence (%) **f**) Depression prevalence (%) **g**) Smoking prevalence (%) **h**) PM2.5 concentration (ug/m3) **i**) O3 concentration (ppb) **j**) Mean temperature (°C) **k**) NDVI (Normalized difference vegetation index).

Figure 4 shows the results of the local feature importance analysis. Poverty and minority determinants are most important in the Northern and Southwest counties, with poverty also important in the Southeast counties. Limited language proficiency is an important determinant of asthma prevalence in Southwest counties, and the uninsured population in Northern counties may contribute to the risk of asthma. In terms of underlying health conditions, obesity prevalence is less important in the Midwest but significant in the Southwest. Meanwhile, the depression prevalence is most pronounced in Western counties and a few Midwest and Northeast counties. Smoking, like obesity, is most important in the Southwest regarding population behavioral disorders. Although PM_2.5_ and O_3_ concentrations are more significant in the Northeast, PM_2.5_ is also more significant in the North and South Midwest and O_3_ is more important in the West and Southwest counties. In western counties, NDVI is a significant predictor of asthma prevalence, along with O_3_. Air temperature is particularly important in explaining the prevalence of asthma in Midwest counties.

## Discussion

### Main findings

This cross-sectional study investigated the prevalence of asthma at the county level in the United States using explainable geospatial machine learning. GWRF outperformed the conventional RF model in terms of the cross-validated prediction error. We found strong indications that asthma-environment associations are non-linear, likely not adequately captured through linear models. Asthma prevalence was, for example, positively associated with poverty, minorities, and green space. Similarly, we observed positive associations with obesity, depression, and smoking. In terms of the variable importance, our results suggested that behavioral disorders (e.g., smoking) and socioeconomic determinants (e.g., poverty) play a more critical role than environmental characteristics (e.g., green space and air pollution). Furthermore, the local importance of these determinants showed remarkable geographic variation suggesting that asthma prevention programs to be effective should be tailor-made for specific areas at risk.

### Interpretation of the results

#### Smoking prevalence

The positive non-linear association between asthma and smoking showed the importance of smoking as a risk factor. Tobacco use affects an estimated 30.8 million adults in the United States [51]. Smoking has been associated with more severe symptoms and hospitalizations and a lower response to treatment in asthmatic patients [74]. Thomson et al. [75] argue that around half of adults with asthma globally are current or former smokers.

When specific determinants co-occur, an area is more likely to develop as a health risk zone. While smoking is a factor in the Arizona asthma epidemic, poverty and O_3_ are also factors. According to Drope et al. [76], tobacco use, and disease burden are increasing among the low-income population. Similarly, while smoking is a significant contributor to the asthma rate in California, 16.6% of adults in the state currently have asthma. However, the California state quitline invests $3.04 per smoker, compared to the national average of $2.28 [77]. Thereby, while in western counties, where smoking has an importance on asthma prevalence, other factors with the strongest importance of PM_2.5_ and O_3_ and obesity may exacerbate asthma prevalence [78]. Furthermore, language proficiency in southwestern counties with a high minority population (32%) [47], where smoking is most prevalent, may contribute to the risk of asthma by preventing accurate diagnosis through communication barriers. The inability to communicate effectively with a healthcare provider restricts patient access, undermines trust in the quality of medical care received, and reduces the likelihood that patients will receive appropriate follow-ups [79]. Furthermore, the number of cigarettes smoked in regions is related to the severity of asthma risk, according to the linear regression [54]. However, researchers do not have an accurate estimate of the number of cigarettes smoked in relation to the prevalence of moderate to severe asthma. The non-linear associations help to break this generalization and consider that other social and environmental factors, in addition to the number of cigarettes smoked, may affect asthma rates [80]. In fact, while the association may be strictly linear in some areas, it may not be in others.

#### Depression prevalence

The overall predominately positive non-linear asthma-depression association shows how vital depression is as an underlying health condition. Approximately 21 million American adults (8.4% of people aged ≥ 18) had a mood disorder (e.g., depressive disorder, dysthymic disorder, and bipolar disorder) in 2020 (CDC, 2020c). However, the association between depression and asthma is not well understood. Our findings highlight the importance of mental health screening for people with asthma and the need for health professionals to alleviate psychological distress in asthma management. While it appears logical that having more severe asthma would be associated with an increased risk of depression, studies have yielded conflicting results. Urrutia et al. [81] and Caulfield [55] found that depressive disorder was common in asthma patients and was associated with increased asthma symptom burden and poor health-related quality of life. However, Janson et al. [82] did not find this association. Because the association between depression and asthma is not straightforward, a non-linear link can help explain it better. Meanwhile, the study suggested by Sagmen et al. [83] that depression and anxiety symptoms, as well as strategies for coping with stress, should be assessed in order to improve asthma control in clinical practice. Moreover, areas where asthma and depression co-occur are more likely to be obese [84], such as southwestern counties in Arizona. Additionally, adults in the west (California) who are exposed to poor air quality and suffer from poverty and depression may be at risk of developing severe asthma. The co-occurrence of diseases, exposures, and social vulnerabilities necessitates the implementation of multiple policies in this regard.

#### Poverty

We found a positive non-linear association between asthma and poverty. Asthma is most common in poor people in the USA [85]. Poverty has the greatest proportionate importance per census county in the north Midwest (North Dakota, South Dakota), southwest (Arizona), and along southeast counties, while it has the least in the rest of the United States. The poor, with the highest asthma prevalence, live in neighborhoods that frequently lack access to basic services (e.g., clean water, sanitation, and healthcare resources) [86]. Living in such an environment exposes the poor to various pathogens from an early age, including viral respiratory infections and high environmental irritants. Many factors are likely to play a role in developing asthma and disease exacerbations [87]. Financial barriers may prevent the poor from receiving appropriate care and limit their ability to purchase medication and access routine healthcare [48]. Poor urban facilities (e.g., access to sports equipment) and urban infrastructure (e.g., well-designed pedestrians, access to green space) obstruct a healthy lifestyle, and participation in physical activities exposes the population to psychological and physical stresses that increase asthma risk [88]. Policymakers and planners should consider identifying disease-burdening elements in poor neighborhoods. Deprivation encourages the development of negative habits, which are then passed down through generations.

#### Obesity prevalence

Asthma and obesity were predominantly positive and non-linear associated. However, the association was inverse asthma prevalence was low. These findings align with Wong et al. [89] and Shailesh and Janahi [90], who reported that obesity impairs lung airway function in asthmatics, leading to increased inflammation. Obesity’s systemic inflammatory reactions cause metabolic, cardiovascular, and respiratory problems. Obesity is a major risk factor for the onset of asthma and contributes significantly to the disease’s severity [22]. Nearly 60% of U.S. adults with severe asthma were obese in 2020 [2]. Obesity prevalence has the greatest proportionate importance per census county in the northern counties of North Dakota, South Dakota, and Minnesota, southwest counties (i.e., Arizona), and southeast counties (i.e., Florida), while it was of least importance in the rest of the country. Instead of designing practices and policies based on the likelihood of asthma exposure in healthy individuals, policymakers and health professionals should consider the underlying health conditions, social vulnerability (e.g., poverty), and PM_2.5_ and O_3_ in southwest counties that may trigger asthma incidence.

#### Other socio-physical and environmental determinants

Our findings indicate that areas with a high proportion of uninsured people are likely to have a prevalence of asthma. Furthermore, our results show that some areas with high asthma prevalence have relatively low uninsured populations. Accordingly, other social and physical determinants should be investigated in those areas to investigate the causes of the high asthma rate. Additionally, the frequency of healthcare visits should be a criterion for exploring the impact and importance of insurance on asthma rates. Although the purpose of our study was not to determine how health insurance improves asthma care, our findings shed light on potential mechanisms. In any case, several studies have emphasized the significance of insurance to control asthma, particularly, they suggested Asthma Health Care Program [50, 91]. The studies (e.g., [92]) found that individuals with severe permanent asthma may be unable to obtain health insurance or that the policy that supports them is prohibitively expensive, affecting asthma prevalence. Meanwhile, our findings show that association between asthma and minority population grows exponentially in a non-linear trend, which is consistent with studies that found no direct positive relationship between asthma and minority population [49]. Asthma outcomes vary geographically; it can be either a non-minority or minority population affected by the respiratory disease due to social and physical living conditions [49]. However, studies (e.g., [93]) show that minority groups are more likely to live in unhealthy environments with limited access to resources, which increases the risk of asthma.

Environmental factors such as outdoor air pollution or dust mites can trigger an asthma attack [94]. Air pollution is one of the world’s largest known environmental health threats and a significant cause of respiratory mortality and morbidity [95]. Several studies address air pollution, particularly PM_2.5_ and O_3_, as major causes of asthma [96]. While in our study PM_2.5_ had an inverse relationship with asthma prevalence predominately. However, when the asthma rate is low in some areas, it has a positive relationship with rising asthma rates. We found that the PM_2.5_ importance on the risk of asthma varies among areas with moderate-to-severe asthma prevalence’s, while the significance of PM_2.5_ is not evident in areas with severe asthmatic patients. According to previous studies ([93]), exposure to PM_2.5_ shows its impacts on respiratory disease in the long-term; however, our findings lack a longitudinal approval on the effects of PM_2.5_ on asthma risk. Similarly, the O_3_ association was mixed, partly linear and partly non-linear. Its importance varies among areas with moderate-to-severe asthma. Meanwhile, our findings show that areas with low O_3_ does not show association with the asthma prevalence. Accordingly, the studies (e.g., [97]) emphasize the importance of investigating the effects of air quality on respiratory disease within the defined term/episodes in which people are exposed to air pollutants, despite the fact that asthmatic people are sensitive to any measure and episode of air pollutants [98]. Hence, we propose that studies on respiratory diseases be divided into two categories: first, the effects of air pollutants on healthy individuals who may develop asthma, and second, the effects of air pollutants on people who are already asthmatic to better control respiratory disease rate.

The predominantly positive association between NDVI and asthma prevalence has revealed that green space is significant in most areas across the United States. However, it is not among the critical variables to explain asthma prevalence. The effects of green space on respiratory health and allergy are limited, and the results vary depending on whether the person lives in urban or rural areas [99]. Additionally, exposure to and interaction with green spaces and biologically diverse environments are associated with physical and mental health benefits [100]. Several studies show that green spaces influence the incidence of asthma and allergies [59, 101]; however, some provide mixed results [23]. This could be due to the heterogeneity of the study settings, as it likely depends on, for example, how, where, and when green space was assessed, as well as other factors that may influence disease incidence (e.g., pollen season). In addition to the area’s size, the green spaces’ structure and characteristics appear important for developing asthma and allergies [102]. One advantage is improved air quality, as increased green spaces of all types of filter harmful particles and substances such as CO_2_ and NO_2_ from the air might reduce asthma and allergy prevalence [103].

The association between temperature and its effects on respiratory health has gained public attention [60]. While the literature significantly associates the temperature drop with asthma prevalence [16, 104, 105], our results evidenced an inverse relationship between asthma and temperature. Overall, the effects of physical determinants on asthma prevalence should be considered in conjunction with social determinants of health, such as poverty, to investigate the intensity of asthma rate per co-occurrence of determinants.

### Strengths and limitations

As we are aware, our study is one of the first to use spatial machine learning to assess the association and co-occurrence of disease, environmental determinants, and social vulnerability in asthma epidemiology. Our data-driven study benefitted from the flexibility of the GWRF to examine non-linearities and variable interaction. While methodologically innovative, a key strength of our model was that the model explicitly assessed spatial heterogeneity, an aspect largely ignored in earlier studies [23]. Relatedly, as the comparison between RF and GWRF has demonstrated, we successfully removed spatial patterns in the model residuals, which otherwise possibly biased the results. Furthermore, this study, among initiative studies, employs interpretable machine learning models that provide a spatial dimension that helps better understand the impact and performance of the variables across the study area [106].

Notwithstanding these strengths, some limitations must be acknowledged when interpreting the findings. While our results may be sensitive to the underlying analytical scale, causal inference is hampered by our data’s cross-sectional and ecological nature. The outcome variable was collected using a telephone survey which likely faces problems due to recall bias and social desirability bias [107]. Furthermore, we did not have access to the person-level raw data, and BRFSS performed a person-level weighting and aggregation of data on a county level. Although several previous research ([88, 108]) utilizing similar data did not include area-level survey weights, we indicate this as a study limitation. In addition to the data limitation, the GWRF implementation had some drawbacks. For example, we searched for optimal hyperparameters using a random grid search, which did not guarantee that we found the most appropriate setting. In some parts of the United States (e.g., counties in the south and west), the *R*^*2*^ was moderately high. In these areas, only a fraction of the variance of the outcome variable was explained (about 50%). Alternative ones should be included in the future to improve the model’s explanatory power in these regions. Other optimization methods for tuning hyperparameters, such as bandit-based algorithms, should be investigated as well. To save time and resources, poor performing hyperparameter configurations are removed in each iteration of these algorithms [109]. The machine learning model's hyper-parameters must be tuned efficiently and accurately to improve its practical application. Lastly, the results should only be interpreted at the county level, and not any other spatial granularity. Ecological fallacy prevents an interpretation on the individual level. Furthermore, we cannot rule out the possibility that our findings are not affected by the spatial scale and zoning used (i.e., the modifiable areal unit problem).

## Conclusion

This paper demonstrates that multiple socio-physical determinants likely explain county-level asthma prevalence in the United States. Utilizing explainable geospatial machine learning, we found that poverty, minority, depression prevalence, obesity prevalence, smoking prevalence, and green space were positively and non-linearly associated with asthma prevalence, while limited language, uninsured, mean temperature, PM_2.5_ and O_3_ were inversely associated. Further, our nationwide assessment of the variable importance indicated that smoking prevalence and depression prevalence were the two most relevant, while green space and limited language were the least. However, notable geographical differences were observable when feature importance was assessed locally. Tackling asthma risk factors through specific health policies is challenging, but we advise that interventions carefully incorporate the co-occurrence of multiple socio-physical determinants and are tailor-made for particular areas.

### Supplementary Information

**Additional file 1: Table S1.** Correlation Between Variables. **Fig S1.** Local Determinant (OUT-OF-BAG R^2^) of GWRF Model: a. red areas explaining OUT-OF-BAG R^2^ (> 0.5), b. red areas explaining OUT-OF-BAG R^2^ (>0.8).

## Data Availability

The datasets used and/or analysed during the current study are available from the corresponding author (Benyamin Hoseini) on reasonable request.
