# Supplementary material for: Socioeconomic and environmental determinants of asthma prevalence: a cross-sectional study at the U.S. County level using geographically weighted random forests
Source: Int J Health Geogr. 2023 Aug 10;22:18. doi: 10.1186/s12942-023-00343-6 (PMC10413687; doi:10.1186/s12942-023-00343-6)
Supplement: Supplementary file 1 — Additional file 1: Table S1. Correlation Between Variables. Fig S1. Local Determinant (OUT-OF-BAG R2) of GWRF Model: a. red areas explaining OUT-OF-BAG R2 (> 0.5), b. red areas explaining OUT-OF-BAG R2 (>0.8). [file 12942_2023_343_MOESM1_ESM.docx]

**Additional file**

**Table S1.** Correlation Between Variables.

|  | **Poverty** | **Limited Lang.** | **NDVI** | **Smoking Prev.** | **Depression Prev.** | **Obesity Prev.** | **PM_2.5_** | **Uninsured** | **Minority** | **Temperature** | **O_3_** | **Asthma Prev.** |
| --- | --- | --- | --- | --- | --- | --- | --- | --- | --- | --- | --- | --- |
| **Poverty** | 1.00 | -0.01 | -0.19 | 0.62 | 0.37 | 0.56 | 0.17 | 0.46 | 0.35 | 0.40 | -0.22 | 0.64 |
| **Limited Lang.** | -0.01 | 1.00 | -0.31 | -0.43 | -0.36 | -0.16 | 0.13 | 0.30 | 0.58 | 0.26 | 0.15 | -0.26 |
| **NDVI** | -0.19 | -0.31 | 1.00 | 0.14 | 0.10 | 0.02 | 0.02 | -0.49 | -0.50 | -0.45 | -0.03 | 0.08 |
| **Smoking Prev.** | 0.62 | -0.43 | 0.14 | 1.00 | 0.65 | 0.65 | 0.12 | 0.22 | -0.16 | 0.22 | -0.31 | 0.68 |
| **Depression Prev.** | 0.37 | -0.36 | 0.10 | 0.65 | 1.00 | 0.27 | 0.20 | 0.06 | -0.30 | 0.11 | -0.21 | 0.65 |
| **Obesity Prev.** | 0.56 | -0.16 | 0.02 | 0.65 | 0.27 | 1.00 | 0.21 | 0.31 | 0.18 | 0.37 | -0.25 | 0.43 |
| **PM_2.5_** | 0.17 | 0.13 | 0.02 | 0.12 | 0.20 | 0.21 | 1.00 | 0.05 | 0.21 | 0.45 | -0.20 | 0.12 |
| **Uninsured** | 0.46 | 0.30 | -0.49 | 0.22 | 0.06 | 0.31 | 0.05 | 1.00 | 0.49 | 0.50 | -0.08 | 0.17 |
| **Minority** | 0.35 | 0.58 | -0.50 | -0.16 | -0.30 | 0.18 | 0.21 | 0.49 | 1.00 | 0.55 | -0.02 | 0.04 |
| **Temperature** | 0.40 | 0.26 | -0.45 | 0.22 | 0.11 | 0.37 | 0.45 | 0.50 | 0.55 | 1.00 | -0.30 | 0.10 |
| **O_3_** | -0.22 | 0.15 | -0.03 | -0.31 | -0.21 | -0.25 | -0.20 | -0.08 | -0.02 | -0.30 | 1.00 | -0.09 |
| **Asthma**  **Prev.** | 0.64 | -0.26 | 0.08 | 0.68 | 0.65 | 0.43 | 0.12 | 0.17 | 0.04 | 0.10 | -0.09 | 1.00 |

| 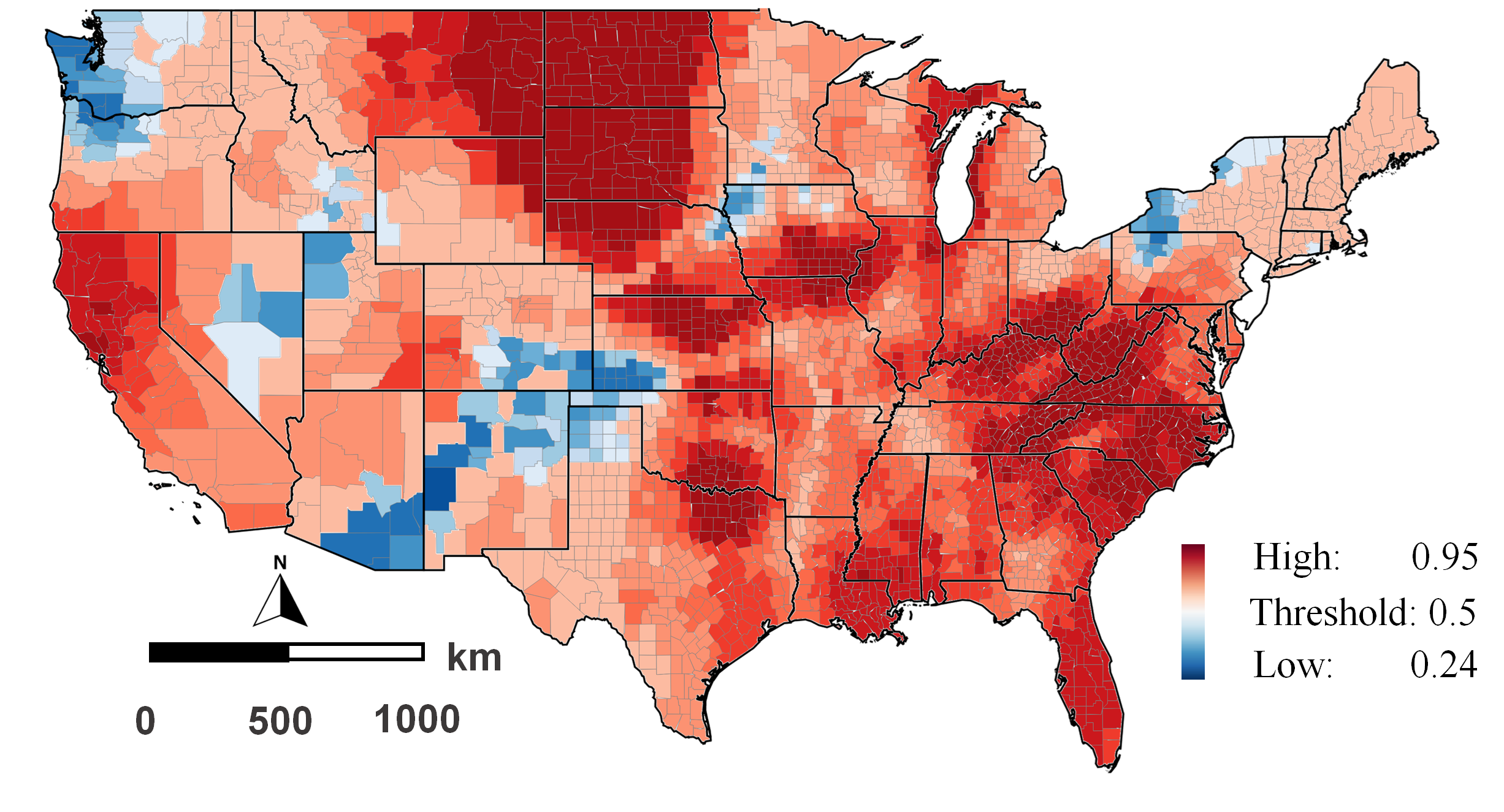 | 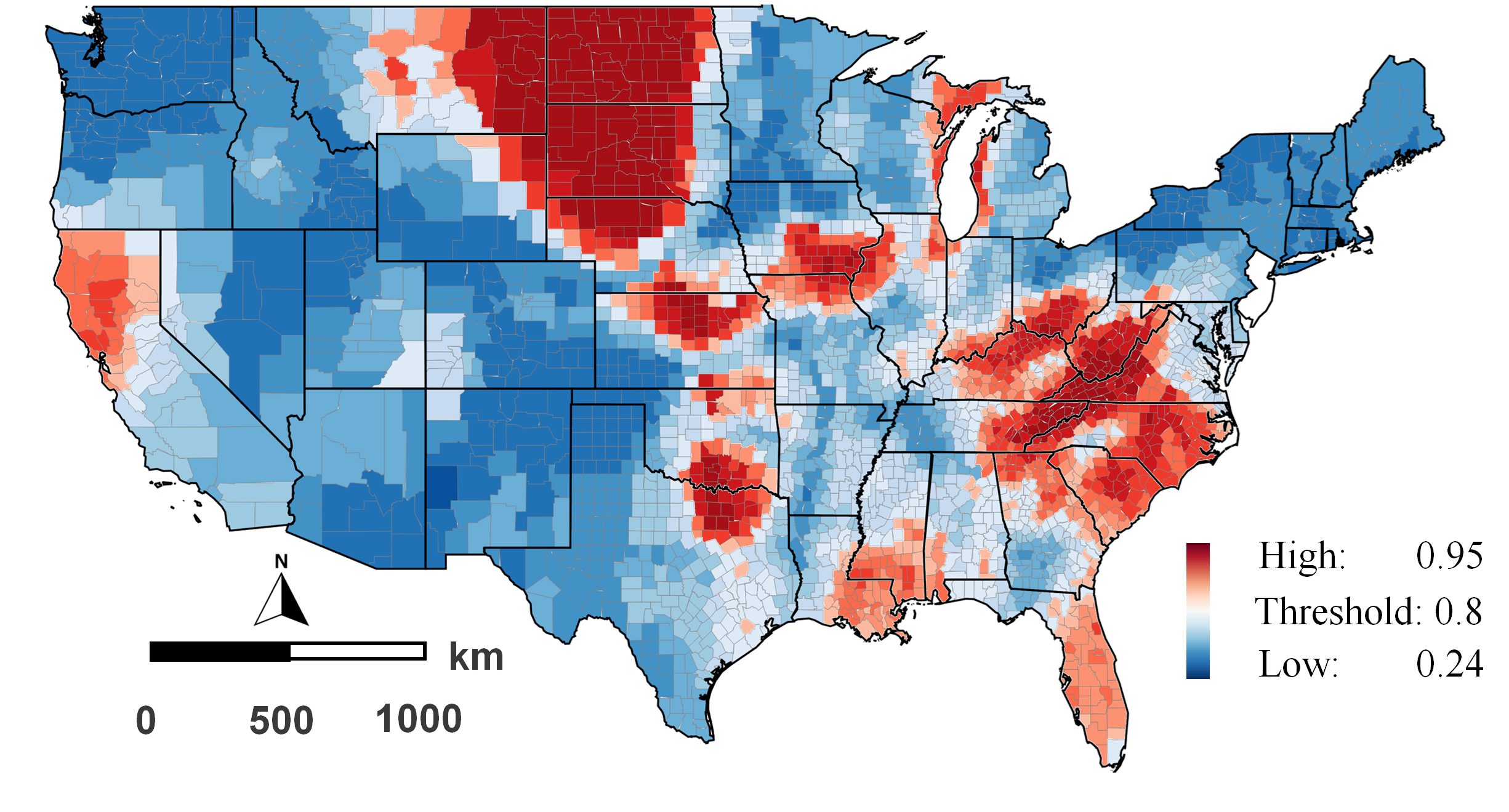 |
| --- | --- |

**Fig S1.** Local Determinant (OUT-OF-BAG R^2^) of GWRF Model: a. red areas explaining OUT-OF-BAG R^2^ (> 0.5), b. red areas explaining OUT-OF-BAG R^2^ (>0.8).

**Additional file Text**

Survey design weights are sometimes used to adjust a possible sample imbalance. The BRFSS employs iterative proportional fitting, or "raking," to account for demographic disparities between the sampled individuals and the population they represent. As a result, the weighting approach is divided into design weight and raking.

The design weights are calculated using the weight of each geographic stratum (STRWT), the number of landline phones within a household (NUMPHON2), and the number of adults who use those phones (NUMADULT). For cell phone respondents, both NUMPHON2 and NUMADULT are set to 1. The formula for the design weight is as follows:

Design Weight = STRWT * (1/NUMPHON2) * NUMADULT

The stratum weight (STRWT) accounts for differences in the probability of selection among strata (subsets of area code/prefix combinations). It is the inverse of the sampling fraction of each stratum.

Then to calculate final weight, BRFSS rakes the design weight to eight margins (gender by age group, race/ethnicity, education, marital status, tenure, gender by race/ethnicity, age group by race/ethnicity, and phone ownership). In addition, BRFSS uses weight trimming to increase the value of extremely low weights and decrease the value of extremely high weights. The objective of weight trimming is to reduce errors in the outcome estimates caused by unusually high or low weights in some categories [50].
